# Adherence trajectories to nicotine replacement therapy among people living with HIV in Uganda: A secondary analysis of the Quit4Life**+** randomized controlled trial

**DOI:** 10.18332/tpc/226097

**Published:** 2026-10-08

**Authors:** Ezekiel Musasizi, David Guwatudde, Elizeus Rutebemberwa, Jim Arinaitwe, Ronald Kusolo, Lynn Atuyambe, Kyra Guy, Heather Wipfli

**Affiliations:** 1 Centre for Tobacco Control in Africa, School of Public Health, Makerere University, Kampala, Uganda; 2 Department of Health Policy, Planning, and Management, School of Public Health, Makerere University, Kampala, Uganda; 3 Department of Epidemiology and Biostatistics, School of Public Health, Makerere University, Kampala, Uganda; 4 Department of Community Health and Behavioral Sciences, School of Public Health, Makerere University, Kampala, Uganda; 5 Department of Preventative Medicine, Keck School of Medicine, University of Southern California, Los Angeles, United States; 6 School of Public Health, University of Maryland, College Park, United States

**Keywords:** nicotine replacement therapy, adherence, people living with HIV, SMS support, Uganda

## Abstract

**Introduction:**

Nicotine replacement therapy (NRT) is an evidence-based approach to tobacco cessation, yet little is known about adherence to NRT among people living with HIV (PLWH) in Sub-Saharan Africa. This study aimed to identify NRT adherence trajectories among PLWH in Uganda and examine both baseline predictors and the role of SMS-delivered support in shaping adherence over time.

**Methods:**

We conducted a secondary analysis of longitudinal data from 200 PLWH, aged ≥18 years with urine cotinine-verified tobacco use and randomized to receive NRT in the Ugandan sites (West Nile and Karamoja regions) of the Quit4Life+ trial, conducted between September 2023 and March 2026. NRT adherence was measured at weeks 2, 6, and 10 using self-reported days of NRT-patch (non)-use and using group-based trajectory modeling to identify adherence patterns. Multinomial logistic regression was used to examine the predictors of trajectory membership. Cross-lagged panel models were used to assess the temporal relationship between perceived stay-quit support and adherence.

**Results:**

Three adherence trajectories emerged: a group with constant adherence (64.1%), a group with improving adherence (13.5%), and a group with worsening adherence (22.4%). High nicotine dependence predicted membership in the group with worsening adherence (adjusted relative risk ratio, ARRR=4.40; 95% CI: 1.92–10.04). Greater perceived stay-quit support was associated with lower odds of worsening-adherence (ARRR=0.39; 95% CI: 0.18–0.86), while more positively perceived message tone was associated with improving-adherence (ARRR=6.60; 95% CI: 1.39–31.26). Cross-lagged analyses showed that early stay-quit support predicted subsequent adherence, whereas adherence did not predict later perceptions of support.

**Conclusions:**

Although baseline characteristics can help identify PLWH at risk of suboptimal adherence, the early weeks of treatment emerged as a potentially important window during which perceived SMS support preceded and predicted subsequent adherence. Combining early risk identification with timely, positively framed SMS support may hold promise for sustaining NRT adherence and could inform future adherence interventions.

## Introduction

Tobacco use is more prevalent among people living with HIV (PLWH) compared to the general population^1^. Studies show that tobacco use among PLWH is associated with poorer adherence to antiretroviral therapy (ART)^2,3^, faster HIV disease progression, and the likelihood of early mortality^4^. Beyond HIV-related outcomes, tobacco use among PLWH increases the risk of non-communicable diseases such as cardiovascular disease and malignancies, which now account for a considerable share of illness and death in this population^5^. Modeling analyses from the multinational Strategies for Management of Antiretroviral Therapy (SMART) trial, which enrolled PLWH from 33 countries, estimated that approximately 24% of all-cause mortality among PLWH is attributable to cigarette smoking, a proportion exceeding mortality attributable to several HIV-related causes combined^6^.

Nicotine replacement therapy (NRT) is a first-line pharmacotherapy for tobacco use cessation^7^, and offers a promising tool to reduce the tobacco-related disease burden among PLWH. NRT has well-documented efficacy in the general population, where it roughly doubles the likelihood of successful cessation. Adherence to prescribed treatment is essential for achieving the therapeutic benefits of NRT^8^, as it minimizes withdrawal symptoms and prevents compensatory smoking. Evidence from clinical trials and real-world studies indicates that suboptimal adherence, including inconsistent use and early discontinuation, substantially attenuates NRT effectiveness and contributes to lower quit rates^8-10^. Notably, NRT adherence is not a static trait but rather a dynamic behavior that can change over the course of treatment, encompassing multiple phases. These are initiation, implementation, and eventual discontinuation^11,12^. Some patients, for example, may initiate NRT but taper off prematurely, or exhibit irregular patterns. One-time or aggregate measures of NRT use may therefore obscure important temporal patterns of adherence.

Characterizing how adherence evolves over time is particularly important among PLWH, who often face unique barriers such as stigma^13,14^ that could cause different adherence behaviors. However, despite the clinical importance of NRT adherence, evidence on how adherence changes over time and determinants among PLWH remains very limited, especially in Sub-Saharan Africa. To our knowledge, little work has been done in identifying the distinct trajectories of NRT use among PLWH who smoke. Most prior research has treated adherence in binary terms (adherent vs non-adherent) or as an average rate, which may overlook meaningful subgroups of patients with different adherence profiles^8^. In addition, behavioral support delivered through short message service (SMS) interventions is increasingly being incorporated into smoking cessation programs to encourage quitting^15,16^, yet little is known about whether participants' experiences of SMS support influence adherence to NRT.

This study aimed to identify NRT adherence trajectories among PLWH who use tobacco in Uganda, characterize the profiles of individuals following each trajectory, and examine the temporal relationship between perceived SMS-delivered support and adherence over time. By combining group-based trajectory modeling^17,18^ with cross-lagged panel analyses^19^, this study contributes to understanding both who may be at risk of suboptimal adherence and the potential behavioral mechanisms through which SMS-delivered support may help sustain adherence during treatment.

## Methods

### Study design

We conducted a secondary analysis of data from the Quit4Life+ randomized controlled trial (RCT), conducted between September 2023 and March 2026. The parent trial was implemented in Zambia (Mongu and Chipata provinces) and Uganda (West Nile and Karamoja regions). The present analysis was restricted to the Ugandan sites, where the study team directly oversaw data collection, and where contextual factors relevant to SMS-based support, including language and HIV service delivery, differ from the Zambian sites, making a within-country analysis more interpretable. Using these data, we examined NRT adherence over the 10 week treatment period. The Quit4Life+trial is registered on ClinicalTrials.gov (NCT05487807; Registration date: 27 November 2023). The Quit4Life+ RCT compares the effectiveness of a phone-based tobacco use cessation intervention for PLWH to the current standard of care (brief advice to quit) and NRT. In the trial, participants were assigned to one of the four study arms: 1) standard-of-care (brief provider advice to quit); 2) standard-of-care (SOC) + NRT; 3) SOC + text message support + messaging (SMS); and 4) SOC + text message support + messaging+ NRT. Block randomization was conducted at the individual level to ensure balanced allocation across study arms. Details of the clinical trial have been published elsewhere^20^. Adherence in the trial was assessed as one of the factors that influence the effectiveness of the interventions.

### SMS-based intervention delivery

This analysis included participants randomized to the SOC+NRT and SOC+NRT+SMS arms of the Quit4Life+ trial^20^. Participants in both arms received nicotine patches together with standard-of-care text messages focused on ART adherence and messages reminding and encouraging them to use their nicotine patches as prescribed. Participants in the SOC+NRT+ SMS arm additionally received the Quit4Life+ tobacco cessation intervention, an adapted version of SmokefreeTXT that was culturally tailored for PLWH in Uganda and Zambia through formative focus groups and usability testing with PLWH tobacco users and healthcare providers^21^. The intervention delivered up to 225 automated messages over 6 months, timed according to each participant’s quit journey and covering pre-quit preparation, quit-day support, and post-quit maintenance. The message schedule was pre-specified, anchored to each participant’s target quit date, with message frequency highest around the quit date and during early maintenance.

Message content addressed motivation, coping with cravings and withdrawal symptoms, social support, HIV-specific health concerns, and locally relevant barriers to cessation, including alcohol use as a trigger and using tobacco to curb hunger. A central feature of the intervention was ongoing ‘Stay-quit’ support, delivered through encouraging and positively framed messages that reinforced participants' confidence in their ability to remain tobacco-free, provided reassurance during difficult moments, and offered practical strategies for managing cravings and preventing relapse. Participants could also request immediate support during cravings (CRAVE), negative moods (MOOD), or smoking slips (SLIP)^20^. Of the up to 225 messages, 164 were standard scheduled messages, 33 were prompted check-in questions (e.g. asking whether the participant had quit), and 28 were available on request. Message content was standardized rather than individually tailored, although message flow varied according to participants' quit stage and responses to check-in prompts. Representative message content and full details of the adaptation process are available in the published protocol and adaptation study^20,21^.

### Study sites

The Quit4Life+ trial was conducted in Uganda and Zambia. In Uganda, from which the analysis in this article is based, the study was conducted in the West Nile and the Karamoja regions. In the West Nile, the trial was conducted in Arua District, while in the Karamoja region, the study was conducted in Moroto and Napak Districts. Both regions are in the northern part of Uganda, as described in the Quit4Life+ trial protocol^20^. These two regions were selected because they have the highest rate of tobacco use in the country, yet differ in cultural context and tobacco use practices. Participants were those enrolled in HIV treatment programs at health facilities within these districts. Details of the participating sites are provided in Supplementary file Table 3.

### Study population

The Quit4Life+ study participants consisted of PLWH receiving ART at the study health facilities and randomized to receive NRT. Eligible participants were HIV-positive individuals receiving antiretroviral therapy (ART) at the study facilities, who received NRT patches, aged ≥18 years, and capable of completing the survey in either the local language (Lugbara language or Nga’karimojong) or English. The exclusion criteria for the Quit4Life+ study included individuals who were very ill or had cognitive impairments. Cognitive impairment was assessed using the Standardized Mini Mental-State Examination (SMMSE), with a score <24 indicating exclusion^22^. The tool was administered by trained clinicians in the participant’s preferred language (Lugbara, Nga’karimojong, or English). Cognitive impairment was assessed using the Standardized Mini Mental-State Examination (SMMSE), with a score <24 indicating exclusion^22^.

The Quit4Life+ trial enrolled 400 participants in Uganda (100 per arm). For this manuscript, the analytic sample includes all 200 participants who were randomized to the two trial arms that received NRT, comprising 100 participants in Arm II (standard care plus NRT) and 100 participants in Arm IV (standard care plus NRT and text message support).

### Data collection

Prior to data collection, the quit4Life+ trial baseline and follow-up questionnaires were pre-tested on 10 PLWH at two non-study HIV clinics (one in Karamoja and another in the West Nile region) to ensure clarity and flow, with minor adjustments made accordingly. The finalized tool was administered by 32 trained research assistants, all of whom were clinicians working within HIV clinics at the selected health facilities, to ensure confidentiality. The survey was conducted through face-to-face interviews using the Open Data Kit (ODK) platform on tablets. At the end of each survey, we checked for missing data to ensure completeness and consistency throughout the data collection process.

### Study variables

The dependent variable for the present study was adherence to NRT patches, defined as the participant’s self-reported number of days on which the PLWH used the patches as prescribed by the healthcare worker at each follow-up time point. Adherence was assessed through self-report using a follow-up questionnaire that asked respondents to indicate the number of days they did not apply the patch over the follow-up period. This was validated by the patch count, as the skins of the patches that were used were returned to the healthcare providers at the follow-up visits. This method of assessing adherence has been used in prior studies^23-25^.

Among the independent variables was nicotine dependence. Nicotine dependence was evaluated using two specific scales: 1) The Fagerström test for nicotine dependence (FTND) for those who smoked, as described by Fagerström and Eisenberg^26^, and 2) the Severson 7-item Smokeless Tobacco Dependence Scale (SSTDS) for those who used smokeless tobacco, according to Mushtaq and Beebe^27^. Tobacco use patterns were assessed using the Smoking Timeline Follow-Back (TLFB) procedure, which has been demonstrated to be a reliable and valid measure^28^.

The sociodemographic variables, such as age, sex, marital status, and education level, which were assessed using questions adapted from the Uganda Demographic and Health Survey (UDHS)^29^. Socioeconomic status (SES) was assessed using a wealth index constructed through principal component analysis (PCA) based on ownership of 15 household assets. These included items such as a radio, television, mobile phone, bicycle, motorcycle, livestock, bed, mattress, sofa set, table, lantern, and access to electricity or piped water. This approach has been widely validated for assessing relative household wealth in low-resource settings.

Respondents were also asked questions regarding their clinical characteristics, including ART adherence, reported using four frequency categories: never, sometimes, most of the time, and all the time. This measure served both as a marker of engagement in routine HIV care and as an indicator of broader treatment adherence behaviors, which may influence NRT use. Behavioral risk factors included alcohol use within the past month, classified into yes, no, abstainer, and not applicable, and other substance use, defined as user versus non-user. These variables were incorporated because substance use is strongly associated with altered nicotine metabolism^30^, impaired behavioral self-regulation, and reduced adherence to cessation pharmacotherapies^31^. Details of the variables are provided in Supplementary file Tables 1 and 2.

### Statistical analysis

We conducted descriptive analyses to characterize the study sample overall and by adherence trajectory group. Categorical variables, including sex, marital status, education level, employment status, household wealth index, region, tobacco use characteristics, ART adherence, alcohol use, substance use, nicotine dependence, tobacco product type, and motivation to quit, were summarized using frequencies and percentages. Continuous variables (age, household size, and years since HIV diagnosis) were summarized using means and standard deviations, or medians and interquartile ranges. We assessed differences across adherence trajectory groups using Pearson’s chi-squared tests for categorical variables and one-way analysis of variance (ANOVA) for continuous variables.

To identify distinct patterns of adherence to NRT, we applied GBTM, a specialized application of finite mixture modeling, using a two-step modeling process. Adherence was assessed at three follow-up time points (weeks 2, 6, and 10 of follow-up, following randomization onto the trial arms), and modeled as a count-based outcome. We estimated multiple candidate models, varying both the number of trajectory groups and the polynomial order (linear, quadratic, cubic) used to describe within-group change over time. Our model selection was guided primarily by the Bayesian information criterion (BIC), with less negative values indicating improved model fit, and by classification quality, assessed using entropy. When multiple models demonstrated comparable statistical fit, preference was given to solutions that yielded distinct and clinically interpretable trajectory shapes, while avoiding redundant or poorly separated groups. Based on these criteria, the final model identified three adherence trajectory groups, specified by polynomial orders (2, 1, 2), with good model fit (BIC= −1429.67) and high classification certainty (entropy=0.889). These were labeled according to their observed patterns: constant adherence (relatively stable adherence across follow-up), worsening adherence (declining adherence over time), and improving adherence (improving adherence over follow-up). Trajectory group membership constituted the primary outcome variable for subsequent analyses.

To examine factors associated with adherence trajectory membership, we fitted bivariate multinomial logistic regression models, with trajectory group as the outcome and the constant adherence group as the reference category. Each baseline characteristic was entered separately to estimate unadjusted associations with membership in the worsening or improving adherence groups. Variables examined included sociodemographic characteristics, household factors, HIV-related variables, substance use behaviors, nicotine dependence, tobacco product type, and motivation to quit.

We then conducted multivariable multinomial logistic regression modeling, including covariates selected *a priori* based on theoretical relevance and evidence from previous literature. These included sex, age, education level, household wealth index, household size, region, nicotine dependence, and motivation to quit. Results were expressed as adjusted relative risk ratios (ARRRs) with corresponding 95% confidence intervals.

To examine the role of SMS support, we generated composite measures capturing different dimensions of participants’ experiences with the SMS intervention, including perceived stay-quit support, message tone, craving support, interactive responsiveness, and overall helpfulness. SMS-related variables were first compared across trajectory groups using ANOVA. SMS components that demonstrated associations with trajectory membership were subsequently examined in multinomial logistic regression models adjusted for the same baseline covariates included in the primary multivariable model.

Following the final multinomial regression models, post-estimation analyses were conducted to facilitate the interpretation of key predictors. Adjusted predicted probabilities of belonging to each adherence trajectory group were estimated using marginal standardization while holding other covariates at their observed values. Predicted probabilities were computed separately for each trajectory and presented graphically to illustrate the relationship between selected predictors and adherence trajectory membership.

To further examine the temporal relationship between perceived stay-quit support and adherence, we fitted cross-lagged panel models using adherence data at Weeks 2, 6, and 10. The models simultaneously estimated autoregressive pathways and cross-lagged pathways between stay-quit support and adherence across successive waves. Indirect effects were estimated to evaluate whether early perceptions of stay-quit support influenced later adherence through intermediate adherence behavior. Model fit was assessed using standard structural equation modeling fit indices, including the comparative fit index (CFI), Tucker–Lewis index (TLI), standardized root mean square residual (SRMR), and root mean square error of approximation (RMSEA). Details of the analysis are provided in the Supplementary file Tables 10-12. All statistical analyses were conducted using Stata version 15.0^32,33^. Statistical significance was assessed using two-sided tests at an alpha level of 0.05.

## Results

### Sociodemographic characteristics of participants

A total of 200 PLWH who were randomized to the two Quit4Life+ trial arms that received an NRT patch were included in this analysis. Participants’ ages ranged 18–83 years, with a mean age of 43.0 years (SD=10.4). The majority were male (71.0%), and most came from the West Nile region (70.0%). Table 1 provides more details of sociodemographic characteristics.

**Table 1:** Sociodemographic characteristics of people living with HIV receiving nicotine replacement therapy: a secondary analysis of the Quit4Life+randomized controlled trial, Uganda (West Nile and Karamoja regions), 2023–2026 (N=200)

Characteristics	Category	n (%)
**Sex**	Male	142 (71.0)
	Female	58 (29.0)
**Age** (years), mean (SD)		43.0 (10.4)
**Marital status**	Unmarried	68 (34.0)
	Currently married	132 (66.0)
**Education level**	Below primary	57 (28.5)
	Primary	76 (38.0)
	Secondary and higher	67 (33.5)
**Wealth index**	Low	67 (33.5)
	Middle	67 (33.5)
	High	66 (33.0)
**Region**	West Nile	140 (70.0)
	Karamoja	60 (30.0)
**Years since HIV diagnosis**, mean (SD)		13.3 (10.5)
**ART adherence**	Never	5 (2.5)
	Sometimes	6 (3.0)
	Most of the time	49 (24.5)
	All the time	140 (70.0)
**Alcohol use** (past month)	No	16 (8.0)
	Yes	118 (59.0)
	Abstainer	66 (33.0)
**Other substance use**	Non-user	156 (78.0)
	User	44 (22.0)
**Nicotine dependence**	Low	125 (65.1)
	High	67 (34.9)
**Type of tobacco**	Smoke	137 (68.5)
	Smokeless	35 (17.5)
	Dual	28 (14.0)
**Motivation to quit**	Not at all	2 (1.0)
	Somewhat	29 (14.5)
	Very much	169 (84.5)
**Household size** (persons), mean (SD)		6.1 (3.6)

Wealth index was derived from household assets using principal component analysis and categorized into tertiles. Nicotine dependence was assessed using the Fagerström test for nicotine dependence for smoking and Severson 7-item Smokeless Tobacco Dependence Scale (SSTDS) for smokeless tobacco. Nicotine dependence data were missing for 8 participants (n=192). ART adherence and alcohol use were self-reported.

ART: antiretroviral therapy; HIV: human immunodeficiency virus; NRT: nicotine replacement therapy.

### Adherence patterns to nicotine replacement therapy

Of the 200 participants included in the trajectory model, 192 had sufficient information to be assigned to a trajectory group. Among these, 123 (64.1%) were in a group with constant adherence, 26 (13.5%) were in a group with improving adherence over time, and 43 (22.4%) were in a group with worsening adherence over time, as shown in Figure 1. Supplementary file Table 4 shows that there was no association between membership in the adherence trajectory groups and study arms.

**Figure 1:**
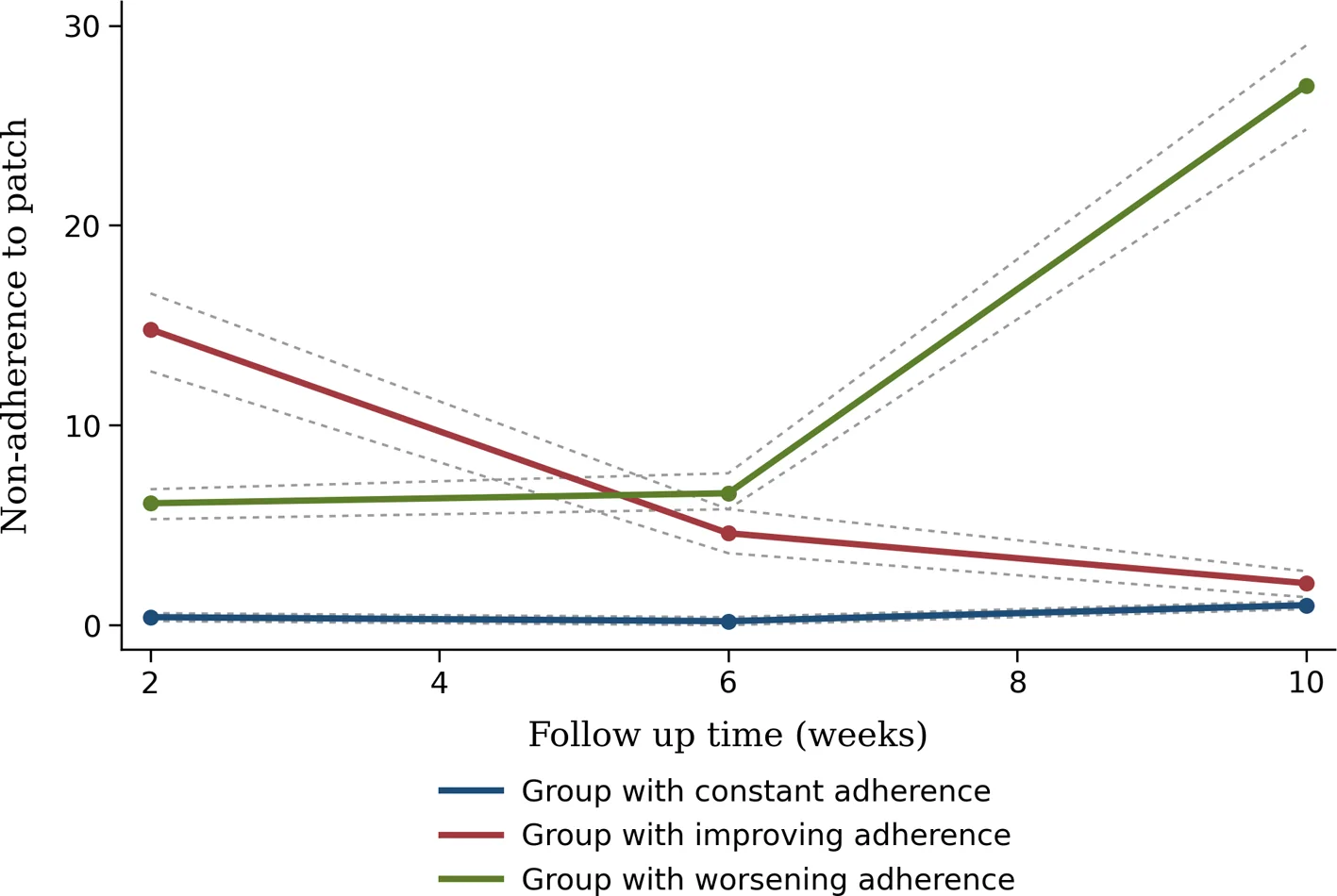
Group-based trajectory model of adherence to nicotine replacement therapy over 10 weeks among people living with HIV: a secondary analysis of the Quit4Life+randomized controlled trial, Uganda (West Nile and Karamoja regions), 2023–2026 (N=192)

### Multinomial logistic regression model

Compared to the group with constant adherence, PLWH in the middle socioeconomic status (SES) group (ARRR=0.29; 95% CI: 0.09–0.93) and those in the high SES group (ARRR=0.20; 95% CI: 0.05–0.74) were less likely to belong to the group with improving adherence over time. Similarly, participants who reported being highly motivated to quit were less likely to belong to the group with improving-adherence compared with constant adherence (ARRR=0.27; 95% CI: 0.08–0.87). In contrast, PLWH with high nicotine dependence were over four times more likely to belong to the group with worsening-adherence (ARRR=4.40; 95% CI: 1.92–10.04) compared with the group with constant adherence, after adjusting for sex, age, education level, household size, and region. Details of the multinomial regression are shown in Table 2.

**Table 2:** Adjusted relative risk ratios for predictors of NRT adherence trajectory membership among people living with HIV: a secondary analysis of the Quit4Life+randomized controlled trial, Uganda (West Nile and Karamoja regions), 2023–2026 (N=192)

Variables	Improving adherencevsconstant adherent group		Worsening adherencevsconstant adherent group	
	ARRR (95% CI)	p	ARRR (95% CI)	p
**Sex**				
Male (ref.)				
Female	0.48 (0.14–1.61)	0.233	1.37 (0.55–3.45)	0.498
**Age** (years)	1.00 (0.95–1.05)	0.931	1.00 (0.96–1.04)	0.956
**Education level**				
Below primary (ref.)				
Primary	1.20 (0.33–4.28)	0.335	0.50 (0.19–1.36)	0.175
Secondary and higher	2.03 (0.51–8.05)	0.311	0.72 (0.26–1.98)	0.522
**Wealth index**				
Low (ref.)				
Middle	0.29 (0.09–0.93)	**0.038**	1.39 (0.55–3.50)	0.488
High	0.20 (0.05–0.74)	**0.016**	0.59 (0.21–1.68)	0.326
**Household size** (persons)	1.00 (0.86–1.16)	0.971	1.05 (0.94–1.17)	0.382
**Region**				
West Nile (ref.)				
Karamoja	1.02 (0.32–3.23)	0.975	1.65 (0.62–4.44)	0.319
**Nicotine dependence**				
Low (ref.)				
High	1.24 (0.44–3.49)	0.685	4.40 (1.92–10.04)	**<0.001**
**Motivation to quit**				
Not/somewhat (ref.)				
Very much	0.27 (0.08–0.87)	**0.029**	1.85 (0.51–6.72)	0.349

Estimates are from a multivariable multinomial logistic regression model including all variables shown, mutually adjusted for one another. The group with constant adherence served as the reference outcome category. Wealth index was derived from household assets using principal component analysis, categorized into tertiles.

ARRR: adjusted relative risk ratio; CI: confidence interval; NRT: nicotine replacement therapy.

### Association between SMS message components and adherence trajectories

After adjustment for sex, age, education level, wealth index, household size, region, nicotine dependence, and motivation to quit, PLWH who reported higher levels of support from the stay-quit SMS messages were less likely to belong to the group with worsening-adherence compared to the group with constant adherence (ARRR=0.39; 95% CI: 0.18–0.86). Conversely, participants who rated the tone of the SMS messages more positively were more likely to belong to the group with improving adherence than to the group with constant adherence (ARRR=6.60; 95% CI: 1.39–31.26). Details are presented in Table 3 and Supplementary file Tables 9 and 10.

**Table 3:** Adjusted relative risk ratios of the associations between SMS components and adherence trajectory membership among people living with HIV: a secondary analysis of the Quit4Life+randomized controlled trial, Uganda (West Nile and Karamoja regions), 2023–2026 (N=192)

Variables	Improving adherence vs constant adherence		Worsening adherence vs constant adherence	
	ARRR (95% CI)	p	ARRR (95% CI)	p
**Stay-quit support**	0.95 (0.41–2.20)	0.911	0.39 (0.18–0.86)	**0.019**
**Message tone**	6.60 (1.39–31.26)	**0.017**	0.56 (0.16–1.99)	0.37

Reference outcome is the group with **constant adherence**. Each SMS component was examined in a separate multinomial logistic regression model adjusted for sex, age, education level, wealth index, household size, region, nicotine dependence, and motivation. Full model results, including all covariate estimates, are provided in Supplementary file Tables 9 and 10.

ARRR: adjusted relative risk ratio; CI: confidence interval.

### Cross-lagged panel model of stay quit support and adherence trajectories

The cross-lagged panel model (Table 4) showed that higher perceived stay-quit support at Week 2 was associated with greater adherence at Week 6 (β=0.311; 95% CI: 0.121–0.502), whereas baseline adherence at Week 2 was not associated with subsequent perceptions of stay-quit support at Week 6 (β=0.010; 95% CI: −0.216–0.235). Adherence at Week 6 was associated with higher adherence at Week 10 (β=0.271; 95% CI: 0.087–0.455). Stay-quit support also demonstrated significant autoregressive effects over time, with support ratings at Week 2 predicting support ratings at Week 6 (β=0.219; 95% CI: 0.050–0.388) and support ratings at Week 6 predicting support ratings at Week 10 (β=0.268; 95% CI: 0.087–0.455). Furthermore, a significant indirect pathway linked early stay-quit support to later adherence through intermediate adherence (unstandardized indirect effect=1.26; 95% CI: 0.13–2.39; standardized indirect effect β=0.084; 95% CI: 0.009–0.160). The corresponding reverse indirect pathway (adherence → stay-quit support) was not significant (standardized indirect effect=0.0003; 95% CI: −0.007–0.008).

**Table 4:** Cross-lagged panel model of Stay-quit support and adherence over three waves(weeks 2, 6, and 10) among people living with HIV: a secondary analysis of the Quit4Life+randomized controlled trial, Uganda (West Nile and Karamoja regions), 2023–2026 (N=192**)**

Path description	Standardized coefficient (β)	95% **CI**	p
**Autoregressive paths (stability over time)**			
Stay-quit support Week 2 → Week 6	0.219	0.050–0.388	0.011
Stay-quit support Week 6 → Week 10	0.268	0.084–0.452	0.004
Adherence Week 2 → Week 6	0.057	−0.168–0.282	0.619
Adherence Week 6 → Week 10	0.271	0.087–0.455	0.004
**Forward cross-lagged paths (stay-quit support → adherence)**			
Stay-quit support Week 2 → Adherence Week 6	0.311	0.121–0.502	0.001
Stay-quit support Week 6 → Adherence Week 10	−0.077	−0.270–0.116	0.436
**Reverse cross-lagged paths (adherence → stay-quit support)**			
Adherence Week 2 → Stay-quit support Week 6	0.01	−0.216–0.235	0.932
Adherence Week 6 → Stay-quit support Week 10	0.168	−0.040–0.376	0.113
**Indirect effects**			
Forward: Stay-quit support Week 2 → Adherence Week 6 → Adherence Week 10	0.084	0.009–0.160	0.029
Reverse: Adherence Week 2 → Stay-quit support Week 6 → Adherence Week 10	0.0003	−0.007–0.008	0.932

Models were adjusted for sex, age, education level, wealth index, household size, region, nicotine dependence, and motivation to quit. Model fit statistics are shown in Supplementary file Table 14.

## Discussion

In this study of PLWH receiving NRT patches through HIV clinics in Uganda, we identified three distinct adherence trajectories over 12 weeks: a group with constant adherence, a group with improving adherence, and a group with worsening adherence. Membership in these trajectories was differentially associated with baseline nicotine dependence, socioeconomic status, and motivation to quit. During follow-up, participants who perceived greater support from the stay-quit SMS messages were less likely to belong to the group with worsening-adherence, whereas those who rated the tone of the SMS messages more positively were more likely to belong to the group with improving-adherence. Furthermore, early perceptions of stay-quit support predicted subsequent adherence, with evidence of a significant indirect pathway linking Week 2 stay-quit support to Week 10 adherence through adherence at Week 6.

We observed that the group with constant adherence accounted for 64.1% of the total sample size. This result is broadly consistent with findings from Browning et al.^24^ who conducted a one-group longitudinal study among HIV-infected smokers in the United States receiving intensive tobacco dependence treatment over a 12 week period and reported that 57.1% of participants completed at least 80% of prescribed pharmacotherapy weeks. In addition to the group with constant adherence, we identified two subgroups of PLWH who exhibited either improving or worsening adherence over time. The group with worsening adherence likely reflects increasing difficulty sustaining treatment in the context of persistent withdrawal symptoms, cravings, and continued tobacco use. Participants in the group with improving adherence showed a progressive increase in NRT use over time, ultimately reaching adherence levels comparable to those observed in the group with constant adherence. This pattern likely reflects a process of treatment familiarization and adaptation, whereby initial challenges related to side effects, patch use routines, or uncertainty about treatment effectiveness diminish with continued use. As such, individuals may experience greater perceived benefit from NRT, reinforcing continued and more consistent use.

We also found that compared with the group with constant adherence, PLWH in the middle- and high-SES categories had significantly lower odds of belonging to the group with improving-adherence, and predicted probabilities showed that consistent adherence increased from low to high SES categories. This pattern suggests that participants with the fewest economic resources may initially struggle with regular patch use, but some are able to catch up over time, whereas wealthier participants either adhere consistently or disengage early. This aligns with extensive evidence that low-SES smokers face more barriers to cessation and are less likely to achieve long-term abstinence, even when they access formal cessation services^34^. Hiscock et al.^34^ showed that smokers from lower socioeconomic groups in England were less likely to quit successfully than more affluent smokers, even within the same stop-smoking services. Our findings are consistent with this SES gradient and suggest that in HIV clinics, NRT adherence support may need to be particularly tailored to address financial stressors that constrain access to NRT among poorer PLWH. Governments should provide NRT free of charge. Clinicians should synchronize NRT refills with routine ART visits, dispensing longer refill intervals, and integrating NRT dispersion into differentiated HIV care delivery models so as to minimize access barriers to NRT among PLWH in the low SES and improve their adherence.

Motivation to quit was another key determinant of adherence patterns. Participants who were highly motivated to quit were less likely to belong to the group with improving-adherence compared to the group with constant adherence, and predicted probabilities showed higher chances of constant adherence among the highly motivated. Motivation is central in the COM-B model, which Mersha et al.^35^ used to synthesize barriers and facilitators of NRT adherence; they concluded that both reflective and automatic motivation (e.g. desire to quit, habits, emotional drivers) are critical in sustaining NRT use. Several other studies have echoed the importance of motivation for quitting tobacco use cessation^36^. Our data are consistent with these findings and highlight that even among PLWH, motivation to quit remains a powerful determinant of how people engage with pharmacological support. This calls for a need to pair NRT with brief, repeated motivational reinforcement (including SMS- or peer-based approaches) to sustain and reinforce motivation over time, rather than relying on a one-time quit message at baseline^23^.

The cross-lagged panel analysis suggests that perceived stay-quit support may be one of the mechanisms through which SMS interventions influence adherence to NRT. Participants who perceived greater stay-quit support early in treatment subsequently demonstrated higher adherence, whereas adherence did not predict later perceptions of support. This temporal ordering argues against the alternative explanation that participants who were already adherent simply evaluated the intervention more positively. Instead, the findings suggest that supportive SMS messages may help shape adherence behavior during the early stages of treatment. The indirect pathway further indicates that participants who perceived greater support at Week 2 were more likely to adhere at Week 6, which in turn predicted adherence at Week 10. This pattern suggests that supportive messaging may contribute to the establishment of adherence habits during the critical initiation phase of NRT use. Once established, these adherence behaviors appear to persist over time, thereby amplifying the long-term benefits of early support. These findings offer a plausible explanation for why participants who perceived greater stay-quit support were less likely to belong to the group with worsening-adherence. SMS messages that are perceived as supportive may help sustain engagement with treatment during periods when adherence is most vulnerable to decline.

### Strengths and limitations

A key strength of this study is the use of GBTM to characterize adherence to NRT among PLWH. Unlike conventional binary adherence measures, this approach revealed substantial heterogeneity in adherence patterns, including groups with constant, improving, and worsening adherence. These findings highlight opportunities for early identification of individuals at risk of worsening adherence and for tailoring adherence support within HIV care.

One limitation of this study is that adherence was assessed within the context of a clinical trial, where participants received structured follow-up and adherence-supportive text messaging. While this may limit direct generalizability to routine care settings, it also enabled detailed assessment of adherence patterns that are rarely captured outside trial contexts. In addition, adherence was primarily measured through self-report and patch counts, without any biological confirmation, which may have led to some misclassification of adherence. Although direct observation of patch use would be the preferred method for measuring adherence, this was not feasible in the present study. To minimize potential bias associated with self-report, participants were asked to return to the clinic with the used patch liners at each follow-up visit. This allowed the study team to verify the number of days on which the patch was used or not used. Similar approaches to assessing NRT adherence using patch counts have been employed in previous studies^23-25^.

## Conclusions

Adherence to NRT among PLWH appears to remain modifiable after treatment initiation. The early weeks of treatment emerged as a potentially important window in which adherence behaviors become established. The perceived stay-quit support preceded and predicted subsequent adherence, rather than the reverse. While the observational nature of these secondary analyses precludes causal conclusions, this temporal ordering strengthens the plausibility of a supportive role for SMS messaging in sustaining engagement with cessation treatment. Efforts to improve adherence to NRT among PLWH should therefore benefit from combining early identification of individuals at risk of suboptimal adherence with behavioral support strategies that reinforce adherence during treatment. These findings could inform the design of trials evaluating such strategies directly. Future research could also examine how SMS cessation support can be embedded into existing ART programs.
