# Supplementary material for: Adherence trajectories to nicotine replacement therapy among people living with HIV in Uganda: A secondary analysis of the Quit4Life+ randomized controlled trial
Source: Tob Prev Cessat. 2026 Oct 8;12:10.18332/tpc/226097. doi: 10.18332/tpc/226097 (PMC13660434; doi:10.18332/tpc/226097)
Supplement: Supplementary file 1 [file TPC-12-60-s001.pdf]

TOBACCO PREVENTION AND CESSATION

Supplementary file

© 2026 Musasizi E. et al.

DOI:
10.18332/tpc/226097

The content has been provided by the author(s) and has not been reviewed, verified, or endorsed by European Publishing. It may not have undergone peer review. The views, opinions, and recommendations expressed are solely those of the author(s) and do not necessarily reflect the position of European Publishing. European Publishing accepts no responsibility or liability for any consequences arising from the use of, or reliance on, this content.

List of Supplementary Tables

Supplementary Table	Title
Supplementary Table 1	Assignment of key study variables
Supplementary Table 2	Assignment of covariates
Supplementary Table 3	Study facilities by region, district and level of care
Supplementary Table 4	Distribution of adherence trajectory groups by trial arm (crude)
Supplementary Table 5	Sociodemographic characteristics by adherence trajectory group (crude)
Supplementary Table 6	Adjusted marginal probabilities of adherence trajectory membership by nicotine dependence, motivation to quit, and wealth index
Supplementary Table 7	SMS component ratings by adherence trajectory group (crude)
Supplementary Table 8	Unadjusted (crude) associations between SMS-related experiences and adherence trajectory membership
Supplementary Table 9	Adjusted association between perceived stay-quit support and adherence trajectory membership
Supplementary Table 10	Adjusted association between perceived message tone and adherence trajectory membership
Supplementary Table 11	Adjusted predicted probabilities of trajectory group membership by level of stay-quit support
Supplementary Table 12	Cross-lagged panel model: stay-quit support and adherence over three waves
Supplementary Table 13	Correlation matrix of SMS component ratings and adherence over three waves
Supplementary Table 14	Fit indices for alternative cross-lagged panel models examining stay-quit support and adherence

Supplementary Table 1. Assignment of key study variables used in the secondary analysis of the Quit4Life+ randomized controlled trial among people living with HIV, Uganda (West Nile and Karamoja regions), 2023–2026 (analytic sample, n = 192).

Dimension	Item	Assignment
Adherence trajectory	Group-based trajectory model using NRT adherence measured at Weeks 2, 6, and 10	1 = Consistently adherent; 2 = Worsening adherence; 3 = Improving adherence
NRT adherence (Week 2)	Number of days NRT was used during the period preceding the Week 2 assessment	Continuous measure (days of NRT use)
NRT adherence (Week 6)	Number of days NRT was used during the period preceding the Week 6 assessment	Continuous measure (days of NRT use)
NRT adherence (Week 10)	Number of days NRT was used during the period preceding the Week 10 assessment	Continuous measure (days of NRT use)
Stay-quit support (Week 2)	"How helpful were the SMS messages in helping you stay quit?"	1 = Not helpful at all; 2 = Slightly helpful; 3 = Helpful; 4 = Very helpful
Stay-quit support (Week 6)	Same question administered at Week 6	1 = Not helpful at all; 2 = Slightly helpful; 3 = Helpful; 4 = Very helpful
Stay-quit support (Week 10)	Same question administered at Week 10	1 = Not helpful at all; 2 = Slightly helpful; 3 = Helpful; 4 = Very helpful
Overall helpfulness	Participant rating of overall helpfulness of SMS messages	Mean score ranging from 1–4; higher scores indicate greater perceived helpfulness
Craving help	Participant rating of how well SMS messages helped manage tobacco cravings	Mean score ranging from 1–4; higher scores indicate greater perceived support

Dimension	Item	Assignment
Stay-quit support	Participant rating of how well SMS messages helped participants remain tobacco-free	Mean score ranging from 1–4; higher scores indicate greater perceived support
Message tone	Participant rating of the tone and wording of SMS messages	Mean score ranging from 1–4; higher scores indicate more favorable perceptions
Interactive responsiveness	Participant rating of perceived responsiveness and interactivity of SMS messages	Mean score ranging from 1–4; higher scores indicate greater responsiveness
Active engagement	Frequency of engaging with or responding to SMS messages	1 = Never; 2 = Sometimes; 3 = Often; 4 = Always

NRT = nicotine replacement therapy; SMS = short message service.

Supplementary Table 2. Assignment of covariates used in the secondary analysis of the Quit4Life+ randomized controlled trial among people living with HIV, Uganda (West Nile and Karamoja regions), 2023–2026 (analytic sample, n = 192).

Dimension	Item	Assignment
Sex	Sex of participant	0 = Male; 1 = Female
Age	Age at enrolment into the trial	Continuous variable (years)
Education	Highest level of education completed	1 = Below primary; 2 = Primary; 3 = Secondary and above
Marital status	Current marital status	0 = Not married; 1 = Married/cohabiting
Employment	Current employment status	0 = Not employed; 1 = Employed
Wealth index	Household wealth index derived from asset ownership indicators	1 = Low; 2 = Middle; 3 = High
Household size	Number of household members living in the household	Continuous variable
Region	Trial region	0 = West Nile; 1 = Karamoja
Tobacco use type	Current form of tobacco use at enrolment	1 = Smoking only; 2 = Smokeless tobacco only; 3 = Dual use
Nicotine dependence	Fagerström Test for Nicotine Dependence (FTND)	1 = Low dependence; 2 = Moderate dependence; 3 = High dependence
Alternative nicotine dependence classification	Combined FTND/STND dependence measure	0 = Low dependence; 1 = High dependence
Motivation to quit	Self-reported motivation to stop tobacco use	Original responses recoded as: 0 = Not motivated/Somewhat motivated; 1 = Very motivated
Alcohol use	Current alcohol use	0 = No; 1 = Yes
Substance use	Current use of other psychoactive substances	0 = No; 1 = Yes
ART adherence	Self-reported adherence to antiretroviral therapy	0 = Non-adherent; 1 = Adherent
Years living with HIV	Time since HIV diagnosis	Continuous variable (years)

FTND = Fagerström Test for Nicotine Dependence; STND = smokeless tobacco nicotine dependence; ART = antiretroviral therapy.

Supplementary Table 3. Study facilities by region, district and level of care, Quit4Life+ randomized controlled trial, Uganda (West Nile and Karamoja regions), 2023–2026 (N = 10 facilities).

Region	District	Health facility	Level of care
West Nile	Arua	Arua Regional Referral Hospital	Regional Referral Hospital
		Adumi Health Centre IV	Health Centre IV

Region	District	Health facility	Level of care
		Omugo Health Centre IV	Health Centre IV
		River Oli Health Centre IV	Health Centre IV
Karamoja	Moroto	Moroto Regional Referral Hospital	Regional Referral Hospital
		Nadunget Health Centre IV	Health Centre IV
		Kosiroi Health Centre II	Health Centre II
		Nakaperimen Health Centre II	Health Centre II
	Napak	Apeitolim Health Centre II	Health Centre II
		Lorengechora Health Centre III	Health Centre III

Supplementary Table 4. Distribution of adherence trajectory groups by trial arm (Arms II and IV) among people living with HIV receiving nicotine replacement therapy: a secondary analysis of the Quit4Life+ randomized controlled trial, Uganda (West Nile and Karamoja regions), 2023–2026 (n = 192).

Trajectory group	Total, n (%)	Arm II, n (%)	Arm IV, n (%)	p-value
Consistant adherence	123 (64.1)	65 (52.9)	58 (47.1)	0.776
Improving adherence	26 (13.5)	9 (34.6)	17 (65.4)	
Worsening adherence	43 (22.4)	23 (53.5)	20 (46.5)	

Values are n (%). Estimates are crude (unadjusted); the p-value was derived from Pearson's chi-square (χ^2) test comparing the distribution of trajectory groups across trial arms.

Supplementary Table 5. Sociodemographic characteristics by adherence trajectory group among people living with HIV receiving nicotine replacement therapy: a secondary analysis of the Quit4Life+ randomized controlled trial, Uganda (West Nile and Karamoja regions), 2023–2026 (n = 192).

Characteristic	Category	Constant adherence (n = 123)	Improving adherence (n = 26)	Worsening adherence (n = 43)	p-value
Sex*	Male	86 (69.9)	19 (73.1)	30 (69.8)	0.946
	Female	37 (30.1)	7 (26.9)	13 (30.2)	
Age, mean (SD)†	–	42.8 (10.7)	41.7 (8.1)	43.6 (10.9)	0.757
Marital status*	Unmarried	44 (35.8)	8 (30.8)	11 (25.6)	0.459
	Currently married	79 (64.2)	18 (69.2)	32 (74.4)	
Education level*	Below primary	34 (27.6)	6 (23.1)	14 (32.6)	0.884
	Primary	49 (39.8)	11 (42.3)	14 (32.6)	
	Secondary and above	40 (32.5)	9 (34.6)	15 (34.9)	
**Wealth index***	**Low**	**37 (30.1)**	**17 (65.4)**	**12 (27.9)**	**0.003**
	**Middle**	**40 (32.5)**	**5 (19.2)**	**20 (46.5)**	
	**High**	**46 (37.4)**	**4 (15.4)**	**11 (25.6)**	
Region*	West Nile	88 (71.5)	14 (53.9)	30 (69.8)	0.206
	Karamoja	35 (28.5)	12 (46.2)	13 (30.2)	
Years since HIV diagnosis, mean (SD)†	–	13.8 (11.5)	11.3 (9.6)	13.3 (7.9)	0.572
ART adherence*	Never	3 (2.4)	0 (0.0)	0 (0.0)	0.528

Characteristic	Category	Constant adherence (n = 123)	Improving adherence (n = 26)	Worsening adherence (n = 43)	p-value
	Sometimes	2 (1.6)	2 (7.7)	2 (4.7)	
	Most of the time	31 (25.2)	7 (26.9)	9 (20.9)	
	All the time	87 (70.7)	17 (65.4)	32 (74.4)	
Alcohol use (past month)*	No	9 (7.3)	1 (3.9)	2 (4.7)	0.562
	Yes	71 (57.7)	19 (73.1)	24 (55.8)	
	Abstainer	43 (35.0)	6 (23.1)	17 (39.5)	
Other substance use*	Non-user	95 (77.2)	20 (76.9)	35 (81.4)	0.840
	User	28 (22.8)	6 (23.1)	8 (18.6)	
**Nicotine dependence***	**Low dependence**	**89 (72.4)**	**17 (65.4)**	**19 (44.2)**	**0.004**
	**High dependence**	**34 (27.6)**	**9 (34.6)**	**24 (55.8)**	
Type of tobacco*	Smoke	84 (68.3)	16 (61.5)	30 (69.8)	0.776
	Smokeless	21 (17.1)	6 (23.1)	7 (16.3)	
	Dual	18 (14.6)	4 (15.4)	6 (14.0)	
**Motivation to quit***	**Not/somewhat**	**16 (13.0)**	**10 (38.5)**	**5 (11.6)**	**0.004**
	**Very much**	**107 (87.0)**	**16 (61.5)**	**38 (88.4)**	
Household size, mean (SD)†	–	5.9 (3.3)	5.4 (2.5)	6.9 (4.5)	0.187

*Values are n (%) unless otherwise indicated. All comparisons are crude (unadjusted). *p-values for categorical variables were derived using Pearson's chi-square (χ^2) test; †p-values for continuous variables were derived using one-way ANOVA. Significant p-values ($p < 0.05$) are shown in bold. ART = antiretroviral therapy; SD = standard deviation.*

Supplementary Table 6. Adjusted marginal probabilities of adherence trajectory membership by nicotine dependence, motivation to quit, and wealth index among people living with HIV receiving nicotine replacement therapy: a secondary analysis of the Quit4Life+ randomized controlled trial, Uganda (West Nile and Karamoja regions), 2023–2026 (n = 192).

Predictor	Category	Consistant adherence		Worsening adherence		Improving adherence		p-value*
		Margin	95% CI	Margin	95% CI	Margin	95% CI	
**Nicotine dependence (FTND)**	Low	0.72	0.64–0.79	0.14	0.08–0.20	0.14	0.08–0.20	0.002
	High	0.49	0.36–0.61	0.39	0.27–0.51	0.12	0.05–0.19	
**Motivation to quit**	Not/Somewhat	0.58	0.39–0.76	0.13	0.01–0.24	0.30	0.12–0.47	0.031
	Very much	0.65	0.58–0.72	0.25	0.18–0.31	0.10	0.05–0.14	
**Wealth index**	Low	0.56	0.43–0.68	0.20	0.10–0.30	0.24	0.13–0.34	0.025
	Middle	0.62	0.50–0.74	0.30	0.19–0.41	0.08	0.01–0.15	
	High	0.76	0.65–0.86	0.17	0.08–0.26	0.07	0.03–0.14	

*Values are adjusted marginal (predicted) probabilities with 95% confidence intervals (CI), estimated from a multinomial logistic regression model adjusted for sex, age, education, wealth index, household size, region, nicotine dependence (FTND), and motivation to quit. *p-value for the joint test of the predictor. FTND = Fagerström Test for Nicotine Dependence.*

Supplementary Table 7. SMS component ratings by adherence trajectory group among people living with HIV receiving nicotine replacement therapy: a secondary analysis of the Quit4Life+ randomized controlled trial, Uganda (West Nile and Karamoja regions), 2023–2026 (n = 192).

SMS component	Constant adherence (n = 116–119)	Worsening adherence (n = 25)	Improving adherence (n = 42–43)	p-value
Overall helpfulness	3.28 (0.25)	3.14 (0.41)	3.36 (0.18)	**0.005**
Craving help	3.72 (0.46)	3.19 (1.03)	3.76 (0.46)	**<0.001**
Stay-quit support	3.68 (0.54)	3.17 (0.97)	3.68 (0.51)	**<0.001**
Message tone	3.59 (0.55)	3.48 (0.64)	3.78 (0.51)	0.068
Interactive responsiveness	3.76 (0.39)	3.57 (0.59)	3.87 (0.32)	**0.019**
Active engagement	1.71 (0.74)	1.83 (0.90)	1.80 (0.66)	0.676

Data are mean (SD). Estimates are crude (unadjusted). Scores range from 1 (lowest) to 4 (highest) for all items except active engagement (1 = Never to 4 = Always). p-values from one-way ANOVA; significant p-values (p < 0.05) are shown in bold. Sample sizes vary slightly due to missing data: constant adherence group n = 119 for overall helpfulness and engagement, n = 116 for other items; improving adherence group n = 43 for overall helpfulness and engagement, n = 42 for other items. SMS = short message service; SD = standard deviation.

Supplementary Table 8. Unadjusted (crude) associations between SMS-related experiences and adherence trajectory membership among people living with HIV receiving nicotine replacement therapy: a secondary analysis of the Quit4Life+ randomized controlled trial, Uganda (West Nile and Karamoja regions), 2023–2026 (n = 192).

SMS component	Worsening vs constant adherence, RRR (95% CI)	p-value	Improving vs constant adherence, RRR (95% CI)	p-value
Overall helpfulness	0.19 (0.05–0.80)	0.024	3.89 (0.85–17.88)	0.081
Craving help	0.35 (0.19–0.64)	0.001	1.21 (0.55–2.65)	0.636
Stay-quit support	0.38 (0.21–0.68)	0.001	1.01 (0.53–1.93)	0.973
Interactive responsiveness	0.44 (0.18–1.05)	0.065	2.55 (0.79–8.19)	0.117
Message tone	0.75 (0.38–1.46)	0.394	2.30 (0.98–5.40)	0.055

RRR = unadjusted (crude) relative risk ratio; CI = confidence interval; SMS = short message service. Estimates are from separate multinomial logistic regression models with each SMS component entered individually and no adjustment for covariates. Reference outcome: constant adherence group.

Supplementary Table 9. Adjusted association between perceived stay-quit support and adherence trajectory membership among people living with HIV receiving nicotine replacement therapy: a secondary analysis of the Quit4Life+ randomized controlled trial, Uganda (West Nile and Karamoja regions), 2023–2026 (n = 192).

Characteristic / category	Improving vs constant adherence		Worsening vs constant adherence	
	aRRR (95% CI)	p-value	aRRR (95% CI)	p-value
Stay-quit support	0.95 (0.41–2.20)	0.911	0.39 (0.18–0.86)	0.019
Message tone	6.60 (1.39–31.26)	0.017	0.56 (0.16–1.99)	0.370
Sex				
Male	Ref.		Ref.	
Female	1.55 (0.50–4.82)	0.446	0.87 (0.20–3.81)	0.855
Age (years)	1.02 (0.97–1.07)	0.508	0.98 (0.92–1.05)	0.601
Education				
Below primary	Ref.		Ref.	
Primary	0.46 (0.16–1.38)	0.168	0.80 (0.20–3.24)	0.755

Characteristic / category	Improving vs constant adherence		Worsening vs constant adherence	
	aRRR (95% CI)	p-value	aRRR (95% CI)	p-value
Secondary and above	0.40 (0.12–1.34)	0.139	0.87 (0.17–4.57)	0.870
Wealth index				
Low	Ref.		Ref.	
Middle	1.06 (0.38–2.99)	0.905	0.42 (0.12–1.48)	0.175
High	0.70 (0.22–2.23)	0.548	0.28 (0.06–1.29)	0.103
Household size	1.07 (0.95–1.20)	0.292	0.98 (0.81–1.18)	0.803
Region				
West Nile	Ref.		Ref.	
Karamoja	1.45 (0.41–5.17)	0.567	1.28 (0.30–5.57)	0.740
Nicotine dependence (FTND)				
Low	Ref.		Ref.	
Moderate	3.80 (1.51–9.55)	0.005	1.42 (0.42–4.77)	0.570
High	2.65 (0.19–36.58)	0.466	2.04 (0.14–30.43)	0.606
Motivation to quit				
Not / somewhat	Ref.		Ref.	
Very motivated	1.79 (0.40–7.91)	0.444	0.81 (0.16–4.00)	0.797

aRRR = adjusted relative risk ratio; CI = confidence interval; Ref. = reference category; FTND = Fagerström Test for Nicotine Dependence. Estimates are from a single multinomial logistic regression model in which all variables shown were entered simultaneously; each aRRR is therefore adjusted for stay-quit support, message tone, sex, age, education, wealth index, household size, region, nicotine dependence (FTND), and motivation to quit. Reference outcome: constant adherence group. Model: LR $\chi^2(24) = 31.27$, $p = 0.146$; pseudo $R^2 = 0.116$.

Supplementary Table 10. Adjusted association between perceived message tone and adherence trajectory membership among people living with HIV receiving nicotine replacement therapy: a secondary analysis of the Quit4Life+ randomized controlled trial, Uganda (West Nile and Karamoja regions), 2023–2026 (n = 192).

Characteristic / category	Improving vs constant adherennce		Worsening vs constant adherence	
	aRRR (95% CI)	p-value	aRRR (95% CI)	p-value
Message tone	6.60 (1.39–31.26)	0.017	0.56 (0.16–1.99)	0.370
Sex				
Male	Ref.		Ref.	
Female	1.41 (0.44–4.54)	0.566	0.80 (0.19–3.36)	0.755
Age (years)	1.01 (0.96–1.07)	0.646	0.97 (0.91–1.04)	0.424
Education				
Below primary	Ref.		Ref.	
Primary	0.39 (0.13–1.19)	0.097	0.70 (0.18–2.73)	0.605
Secondary and above	0.39 (0.12–1.29)	0.122	0.57 (0.11–2.81)	0.486
Wealth index				
Low	Ref.		Ref.	
Middle	0.97 (0.34–2.77)	0.952	0.34 (0.10–1.19)	0.091
High	0.79 (0.24–2.59)	0.695	0.23 (0.05–1.07)	0.061
Household size	1.09 (0.96–1.24)	0.192	0.95 (0.80–1.14)	0.591
Region				

Characteristic / category	Improving vs constant adherennce		Worsening vs constant adherence	
	aRRR (95% CI)	p-value	aRRR (95% CI)	p-value
West Nile	Ref.		Ref.	
Karamoja	1.59 (0.44–5.76)	0.479	1.30 (0.31–5.42)	0.720
Nicotine dependence (FTND)				
Low	Ref.		Ref.	
Moderate	3.69 (1.44–9.45)	0.006	1.31 (0.40–4.33)	0.654
High	2.32 (0.17–31.46)	0.527	4.11 (0.31–54.70)	0.284
Motivation to quit				
Not / somewhat	Ref.		Ref.	
Very motivated	1.45 (0.32–6.66)	0.631	0.94 (0.19–4.58)	0.941

aRRR = adjusted relative risk ratio; CI = confidence interval; Ref. = reference category; FTND = Fagerström Test for Nicotine Dependence. Estimates are from a single multinomial logistic regression model in which all variables shown were entered simultaneously; each aRRR is therefore adjusted for message tone, sex, age, education, wealth index, household size, region, nicotine dependence (FTND), and motivation to quit. Reference outcome: consistently adherent group. Model: LR $\chi^2(24) = 34.77$, $p = 0.072$; pseudo $R^2 = 0.129$.

Supplementary Table 11. Adjusted predicted probabilities of trajectory group membership by level of stay-quit support among people living with HIV receiving nicotine replacement therapy: a secondary analysis of the Quit4Life+ randomized controlled trial, Uganda (West Nile and Karamoja regions), 2023–2026 (n = 192).

Stay-quit support score	Predicted probability of worsening adherence vs constant adherent (95% CI)	Predicted probability of improving adherence vs constant adherent (95% CI)
1 (lowest)	0.374 (0.088–0.660), p = 0.010	0.068 (–0.050–0.186), p = 0.256
2	0.248 (0.096–0.401), p = 0.001	0.125 (–0.003–0.254), p = 0.055
3	0.147 (0.080–0.213), p < 0.001	0.206 (0.110–0.302), p < 0.001
4 (highest)	0.076 (0.028–0.124), p = 0.002	0.307 (0.222–0.392), p < 0.001

Values are adjusted predicted probabilities (95% confidence intervals). Probabilities were calculated from the adjusted multinomial logistic regression model (Supplementary Table 9), adjusted for sex, age, education, wealth index, household size, region, nicotine dependence (FTND), and motivation to quit. Reference outcome: constant adherent group. CI = confidence interval.

Supplementary Table 12. Cross-lagged panel model of stay-quit support and adherence over three waves among people living with HIV receiving nicotine replacement therapy: a secondary analysis of the Quit4Life+ randomized controlled trial, Uganda (West Nile and Karamoja regions), 2023–2026 (n = 192).

Path description	Standardised coefficient (β)	95% CI	p-value
**Forward pathways (hypothesised causal direction)**			
Week 2 stay-quit support → Week 6 adherence	0.311	0.121–0.502	0.001
Week 6 adherence → Week 10 adherence	0.271	0.087–0.455	0.004
Indirect effect (Week 2 support → Week 6 adherence → Week 10 adherence)	0.084*	0.009–0.160	0.029
**Reverse pathways (alternative temporal order)**			
Week 2 adherence → Week 6 stay-quit support	0.010	–0.216–0.235	0.932
Week 6 stay-quit support → Week 10 adherence	–0.077	–0.270–0.116	0.436

Path description	Standardised coefficient (β)	95% CI	p-value
Indirect effect (reverse: Week 2 adherence → Week 6 support → Week 10 adherence)	0.0003	–0.007–0.008	0.932

*Standardised coefficients are from the structural equation model output. *The standardised indirect effect is calculated as the product of the two standardised path coefficients (0.311 × 0.271 = 0.084); the unstandardised indirect effect (1.259, p = 0.029) was also significant. Model fit: $\chi^2(6) = 53.5$, $p < 0.001$; CFI = 0.392; RMSEA = 0.206. CI = confidence interval; CFI = Comparative Fit Index; RMSEA = Root Mean Square Error of Approximation.*

Supplementary Table 13. Correlation matrix of SMS component ratings and adherence over three waves among people living with HIV receiving nicotine replacement therapy: a secondary analysis of the Quit4Life+ randomized controlled trial, Uganda (West Nile and Karamoja regions), 2023–2026 (n = 192).

Variable	1	2	3	4	5	6	7	8	9	10
1. Stay-quit support, W2	1									
2. Stay-quit support, W6	0.19*	1								
3. Stay-quit support, W10	0.42***	0.31***	1							
4. Adherence, W2	0.26**	–0.01	0.22*	1						
5. Adherence, W6	0.25**	0.35***	0.19	0.03	1					
6. Adherence, W10	–0.15	0.04	0.01	0.29**	0.27**	1				
7. Stay-quit composite, avg	0.77***	0.70***	0.83***	0.28***	0.36***	–0.04	1			
8. Message tone	0.19*	0.24**	0.21*	0.00	0.21*	0.00	0.32***	1		
9. Craving help	0.59***	0.45***	0.78***	0.24**	0.23**	–0.07	0.81***	0.31***	1	
10. Interactive responsiveness	0.30***	0.03	0.27**	0.15	–0.01	–0.11	0.34***	0.45***	0.38***	1

*Pairwise correlations *$p < 0.05$, **$p < 0.01$, ***$p < 0.001$. W2, W6 and W10 denote Weeks 2, 6 and 10, respectively. The stay-quit composite is the average of the three wave-specific stay-quit support items; message tone, craving help and interactive responsiveness are overall ratings (single time point). SMS = short message service.*

Supplementary Table 14. Fit indices for alternative cross-lagged panel models examining stay-quit support and adherence among people living with HIV receiving nicotine replacement therapy: a secondary analysis of the Quit4Life+ randomized controlled trial, Uganda (West Nile and Karamoja regions), 2023–2026 (n = 192).

Model	Description	χ^2/df	CFI	TLI	RMSEA	AIC	BIC
M1	Full CLPM + covariates (motivation, FTND, SES)	8.92	0.392	–1.633	0.206	5212.60	5367.70
M2	Full CLPM without covariates	8.47	0.394	–0.413	0.202	3933.52	4000.92
M3	Forward pathway only (Week 2 support → Week 6 adherence → Week 10 adherence)	10.63	0.300	–0.634	0.230	3577.27	3631.74
M4	Simplified mediation model (Week 2 support → Week 6 adherence → Week 10 adherence)	5.13	0.756	0.269	0.153	2315.19	2340.60

CLPM = cross-lagged panel model; χ^2/df = chi-square test of model fit divided by degrees of freedom; CFI = Comparative Fit Index; TLI = Tucker–Lewis Index; RMSEA = Root Mean Square Error of Approximation; AIC = Akaike Information Criterion; BIC = Bayesian Information Criterion; FTND = Fagerström Test for Nicotine Dependence; SES = socioeconomic status (wealth index).
